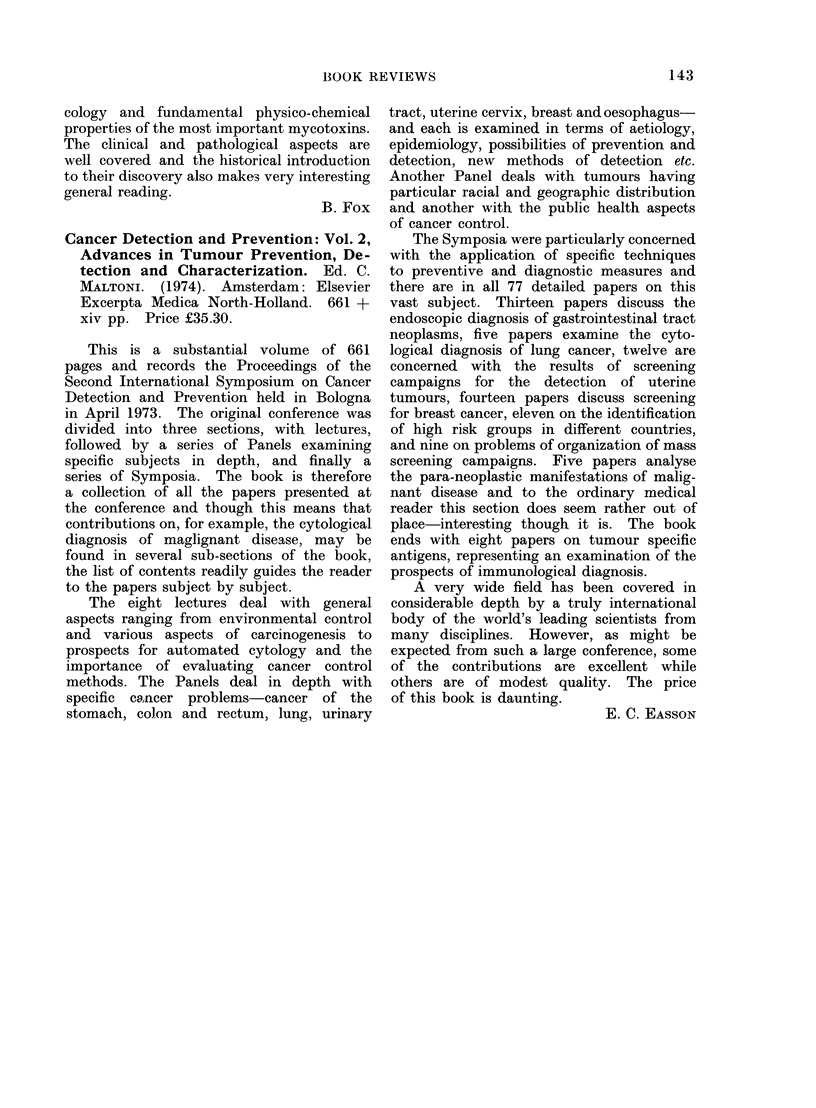# Cancer Detection and Prevention: Vol. 2, Advances in Tumour Prevention, Detection and Characterization

**Published:** 1975-07

**Authors:** E. C. Easson


**Cancer Detection and Prevention: Vol. 2, Advances in Tumour Prevention, Detection and Characterization.** Ed. C. Maltoni. (1974). Amsterdam: Elsevier Excerpta Medica North-Holland. 661 + xiv pp. Price £35.30.

This is a substantial volume of 661 pages and records the Proceedings of the Second International Symposium on Cancer Detection and Prevention held in Bologna in April 1973. The original conference was divided into three sections, with lectures, followed by a series of Panels examining specific subjects in depth, and finally a series of Symposia. The book is therefore a collection of all the papers presented at the conference and though this means that contributions on, for example, the cytological diagnosis of maglignant disease, may be found in several sub-sections of the book, the list of contents readily guides the reader to the papers subject by subject.

The eight lectures deal with general aspects ranging from environmental control and various aspects of carcinogenesis to prospects for automated cytology and the importance of evaluating cancer control methods. The Panels deal in depth with specific cancer problems—cancer of the stomach, colon and rectum, lung, urinary tract, uterine cervix, breast and oesophagus—and each is examined in terms of aetiology, epidemiology, possibilities of prevention and detection, new methods of detection *etc.* Another Panel deals with tumours having particular racial and geographic distribution and another with the public health aspects of cancer control.

The Symposia were particularly concerned with the application of specific techniques to preventive and diagnostic measures and there are in all 77 detailed papers on this vast subject. Thirteen papers discuss the endoscopic diagnosis of gastrointestinal tract neoplasms, five papers examine the cytological diagnosis of lung cancer, twelve are concerned with the results of screening campaigns for the detection of uterine tumours, fourteen papers discuss screening for breast cancer, eleven on the identification of high risk groups in different countries, and nine on problems of organization of mass screening campaigns. Five papers analyse the para-neoplastic manifestations of malignant disease and to the ordinary medical reader this section does seem rather out of place—interesting though it is. The book ends with eight papers on tumour specific antigens, representing an examination of the prospects of immunological diagnosis.

A very wide field has been covered in considerable depth by a truly international body of the world's leading scientists from many disciplines. However, as might be expected from such a large conference, some of the contributions are excellent while others are of modest quality. The price of this book is daunting.

E. C. Easson